# Short-term forecasting of daily infections, fatalities and recoveries about COVID-19 in Algeria using statistical models

**DOI:** 10.1186/s43088-021-00136-5

**Published:** 2021-08-19

**Authors:** Firdos Khan, Mohamed Lounis

**Affiliations:** 1 School of Natural Sciences (SNS), National University of Sciences and Technology (NUST), H-12 Sector, Islamabad, 44000 Pakistan; 2 Department of Agro-Veterinary Science, Faculty of Natural and Life Sciences, University of Ziane Achour, Road of Moudjbara, BP 3117, 17000 Djelfa, Algeria

**Keywords:** Algeria, Time series model, Forecasting, COVID-19, Infections, Recoveries

## Abstract

**Background:**

A viral disease due to a virus called SARS-Cov-2 spreads globally with a total of 34,627,141 infected people and 1,029,815 deaths. Algeria is an African country where 51,690, 1,741 and 36,282 are currently reported as infected, dead and recovered. A multivariate time series model has been used to model these variables and forecast their future scenarios for the next 20 days.

**Results:**

The results show that there will be a minimum of 63 and a maximum of 147 new infections in the next 20 days with their corresponding 95% confidence intervals of − 89 to 214 and 108–186, respectively. Deaths’ forecast shows that there will be 8 and 12 minimum and maximum numbers of deaths in the upcoming 20 days with their 95% confidence intervals of 1–17 and 4–20, respectively. Minimum and maximum numbers of recovered cases will be 40 and 142 with their corresponding 95% confidence intervals of − 106 to 185 and 44–239, respectively. The total number of infections, fatalities and recoveries in the next 20 days will be 1850, 186 and 1680, respectively.

**Conclusion:**

The results of this study suggest that the new infections are higher in number than recover cases, and therefore, the number of infected people may increase in future. This study can provide valuable information for policy makers including health and education departments.

## Background

Since December 2019, the world is facing a new respiratory disease called coronavirus disease 2019 (COVID-19). This viral respiratory illness was first detected in the China’s city of Wuhan in the province of Hubei [1]. On January 30, 2020, the World health organization (WHO) has declared it as an epidemic of "public health emergency of international concern." Forty days later, on March 11th, it has finally recognized this disease as a global pandemic urging all countries to intensify their efforts to prevent its propagation stressing the importance of detection, testing, treatment, isolation, tracing and people mobilization in the anti-COVID-19 response [2]. As the number of affected persons is more than 34 million cases and 1,029,815 deaths through the world until now [3], this disease has attracted a global interest and enormous numbers of researches are in continuous struggle to understand its epidemiological and clinical characteristics. In addition to medical and experimental studies, researchers are using mathematical and statistical models to predict the number of affected persons, the peak and the ending time of the epidemic. The predicted scenarios can be used to assess the preventive measures which could be of great importance for decision-makers to adopt the best strategies in the anti-COVID-19 battle [4].

In this way, multiple models have been proposed for modeling COVID-19 pandemic in different countries including compartmental models, natural growth model and logistic growth models [5]. The SIR (susceptible–infectious–recovered) and SEIR (susceptible–exposed–infectious–recovered) historical compartmental models and their variants are the most used in forecasting human epidemic diseases [6]. They are also widely used in the case of COVID-19 [7]. However, these models have failed so far to give a good description of the empirical data [8]. Time series models (TSMs) are autoregressive moving average models which attempt to predict future events by means of aggregating recent data [8]. These models have shown many successful implementations in economics, finance, climatology, hydrology engineering and epidemiology [9]. The autoregressive integrated moving average (ARIMA), long short-term memory networks (LSTM) are the most commonly used for forecasting epidemic diseases [10]. These models have recently been widely used to forecast COVID-19 epidemic [11–16].

The aim of this study is to identify the data generating process of COVID-19 infections, fatalities and recoveries for Algeria and to forecast future scenarios about these variables. To our knowledge, there is no such study so far to forecast each variable of COVID-19 in Algeria; therefore, the findings of this research may help policy makers to reshape their polices according to the predicted scenario of COVID-19. Further, the results of this study can help various stakeholders including health and education department etc., in making their future’s plans.

## Data and study area

Daily data of new confirmed cases, deaths and recoveries about COVID-19 over Algeria from February 25 to October 1, 2020, were collected form World Health Organization (WHO). In Algeria, the first case of COVID-19 was reported on February 25, 2020. This methodology is applicable to any other region of the world as long as you have time series data.

## Methods

Time series models have multitude of applications in different areas from finance to climatology and hydrology and have shown good performances in forecasting future scenarios [17–19]. There are different classes of time series models, and they can be used to model time series data which depends on their nature. Due to the dependence nature of the considered three variables, it was decided to model these variables jointly. Consequently, VAR model was used for modeling and forecasting COVID-19 in Algeria. Suppose we have a vector of time series denoted by ${Y}_{t},$ then AVR model can be expressed by Eq. (1)

1 $${{\varvec{Y}}}_{{\varvec{t}}}= {{\varvec{B}}}_{0}+ {{\varvec{B}}}_{1}{{\varvec{Y}}}_{{\varvec{t}}-1}+{{\varvec{B}}}_{2}{{\varvec{Y}}}_{t-2}+\dots + {{\varvec{B}}}_{{\varvec{p}}}{{\varvec{Y}}}_{{\varvec{t}}-p}+ {{\varvec{\epsilon}}}_{{\varvec{t}}}$$

where in Eq. (1), ${{\varvec{B}}}_{0}\boldsymbol{ }\mathrm{and }{{\varvec{\epsilon}}}_{{\varvec{t}}}$ are $k\times 1$ column vectors and${\boldsymbol{ }\boldsymbol{ }{\varvec{B}}}_{{\varvec{i}}}$ (for i = 1, 2,..p) is $k\times k$ matrices of coefficients. ${{\varvec{Y}}}_{{\varvec{t}}}$ is a vector of ${\varvec{k}}\times 1$ time series variables. To incorporate VAR model, a four-step methodology have been implemented and given below:

i. model selection (lag length)
ii. estimation of unknown parameters of identified model
iii. diagnostic check of estimated model
iv. forecasting

The first step in modeling a time series data with VAR model is the selection of lags. In the literature, there are some criteria available for this purpose like Hannan–Quinn criterion (HQC) [20], Akaike’s information criterion (AIC) [21], prediction error (FPE) [22] and Schwarz criterion (SC) [23]. The second step consists on the estimation of unknown parameters of identified model in the first step. Among various methods for estimation, ordinary least square (OLS) method of estimation has been used in this study. In the third step, the estimated model is diagnosed by using various statistical and graphical tests. The residuals of the estimated model can be visualized for normality, serial correlation and autoregressive conditional heteroscedasticity (ARCH) error. Statistical tests like normality and serial correlation test can be used for checking the assumption of the estimated model. The final step in time series modeling is to forecast the future’s phenomena on the basis of fitted model to available data. Generally, n-step ahead forecast can be made. However, this depends on the nature and objective of the study. For analysis and visualization, we have used R software with packages mFilter [24] and vars [25].

## Results

**Fig. 1:**
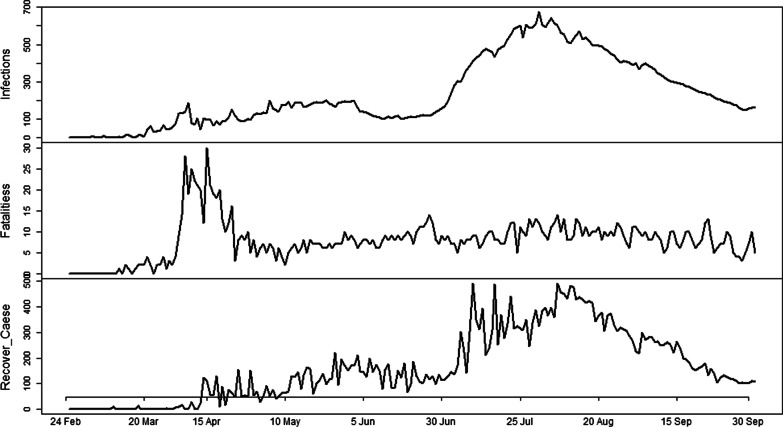
Daily infections, fatalities and recoveries from COVID-19 in Algeria from 24 February to 5 September 2020. Number of infections, fatalities and new recoveries are presented on y-axis while time is mentioned on x-axis

The daily new infections, fatalities and recoveries are displayed in Fig. 1, where it is obvious that there is an increasing trend in different time durations and a decreasing trend in other durations in Algeria. Table 1 presents the correlation structure among **COVID-19** infections, fatalities and recoveries in Algeria. It can be seen that there is a positive relationship among these variables, and therefore, these variables were modeled jointly. VAR model was used to describe the data generating process of all these variables where the lag length was selected by using model selection criteria like AIC, SC, HQC and FPE. The best model was selected with 18 lags. The unknown parameters of identified model were estimated by using OLS method of estimation. The residuals of fitted model were analyzed and checked for normality and serial correlation graphically as well as numerically. It was found that the model has no serial correlation and the residuals follow approximate normal distribution. In addition, the residuals of the fitted model were investigated for ARCH error; however, the null hypothesis of ARCH error was rejected and it was concluded that there is no ARCH error. The fitted model was then used for 20 days ahead forecast of new cases, deaths and recovered cases of COVID-19 in Algeria.

**Table 1:** Dependence structure among daily new cases, deaths and recoveries about COVID-19 in Algeria given by correlation matrix

	Infections	Fatalities	Recoveries
Infections	1.0000	0.3191	0.9206
Fatalities	0.3191	1.0000	0.2876
Recoveries	0.9206	0.2876	1.0000

**Fig. 2:**
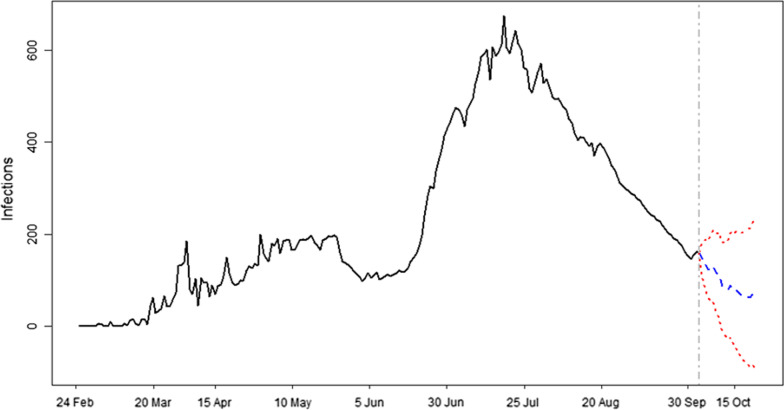
Forecast of new cases of COVID-19 in Algeria. Forecast of infections and time are mentioned on y-axis and x-axis, respectively. Blue color shows the forecast of infections and 95% confidence intervals are represented by red color

**Fig. 3:**
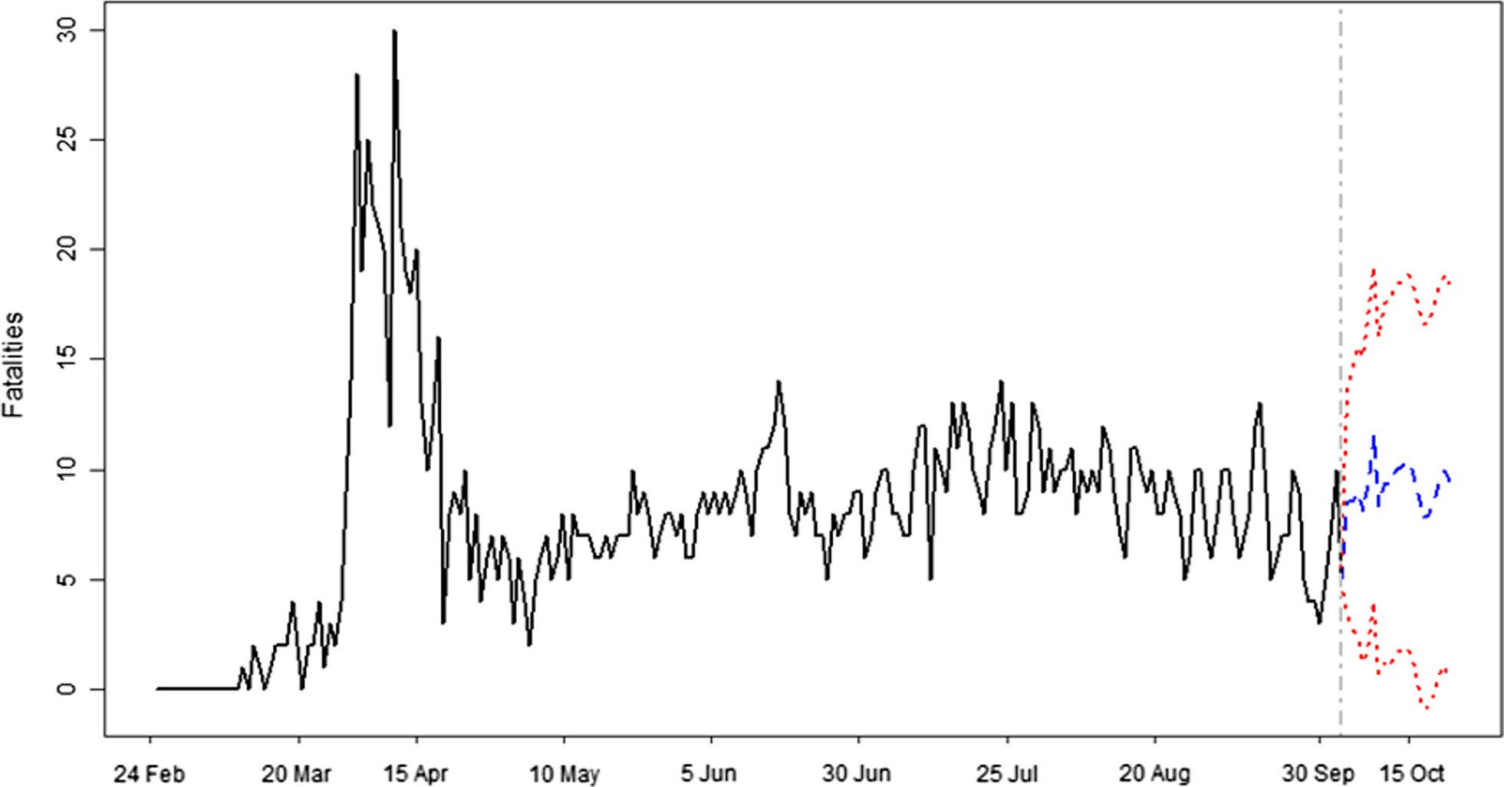
Forecast of daily deaths due to COVID-19 in Algeria. Number of deaths and time are represented on y-axis and x-axis, respectively. Blue color shows the forecast of daily deaths and 95% confidence intervals are represented by red color

**Fig. 4:**
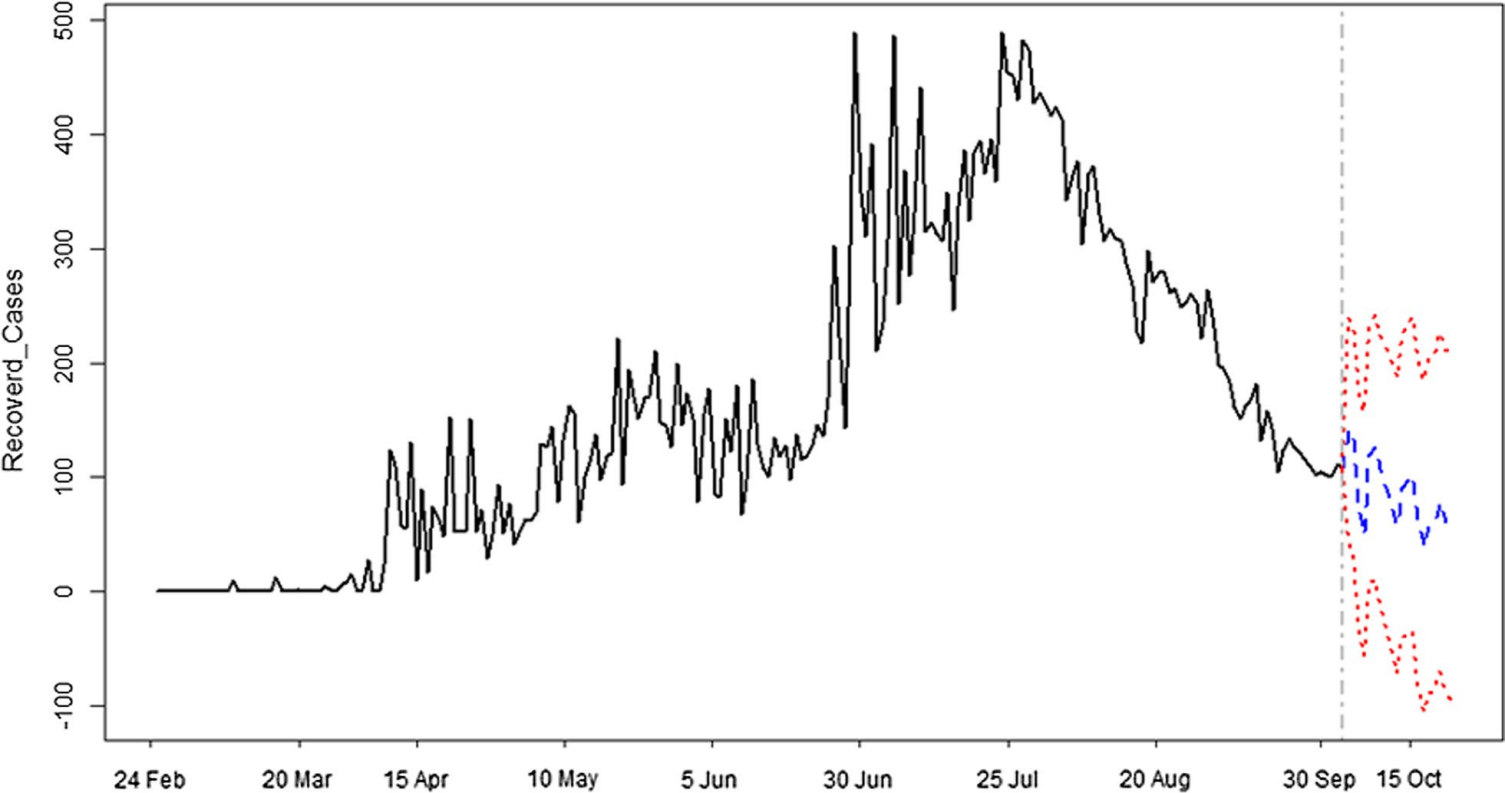
Daily recovered cases from COVID-19 in Algeria. Number of recovered cases and time are mentioned on y-axis and x-axis, respectively. Blue color shows the forecast of recovered cases and 95% confidence intervals are represented by red color

The forecast results are presented in Fig. 2–4 and Table 2 where Figs. 2, 3 and 4 show results for new infections, fatalities and recover cases, respectively. Table 2 presents the forecast results of minimum, maximum and average values with their corresponding 95% confidence intervals for each variable of the forecast duration. The 20 days ahead forecast results for new cases of COVID-19 show that there is a decreasing trend. The maximum number of daily infections will be 147 with 95% confidence intervals of 108–186. The minimum number of daily infections forecast by our model is 63 with 95% confidence intervals of − 89 to 214. Regarding fatalities, results for next 20 days show that their number will varied from 8 to 12 deaths per day, with a corresponding 95% confidence intervals of − 1 to 17 and 4–20, respectively. The model’s forecast results for daily recovered cases show that there will be a minimum of 40 [CI = − 106 to 185] and a maximum of 142 [CI = 44–239] recovered persons per day in the upcoming 20 days in Algeria. On the average, there will be 93, 10 and 84 new infections, fatalities and recoveries with their corresponding 95% confidence intervals of − 16 to 201, 2–18 and − 45 to 210, respectively.

**Table 2:** Forecast values for daily infections, fatalities and recoveries from COVID-19 with their corresponding 95% confidence intervals for Algeria in upcoming 20 days

Variable	Max/Min	Forecast	95% confidence intervals		Total cases in 20 days
			Lower limit	Upper limit	
Infections	Min	63	− 89	214	1850
	Max	147	108	186	
	Average	93	− 16	201	
Fatalities	Min	8	− 1	17	186
	Max	12	4	20	
	Average	10	2	18	
Recoveries	Min	40	− 106	185	1680
	Max	142	44	239	
	Average	84	− 45	210

The forecast results of VAR model for next 20 days show that the forecast values follow the trend of observed data reasonably. It can be seen from the forecast results that the average number of new recoveries is lower than the average number of new infections in next 20 days. Therefore, there will probably be more infected people in Algeria in the future. However, these results based on our model and the number of infected people may be reduced by following the SOPs of WHO and Algerian government about COVID-19.

## Discussion

There are various studies about COVID-19 forecasting in different region and countries of the world [9, 15, 16, 19, 26–28] including Pakistan, Canada, Germany, UK, France, Italy, Russia, Japan and Turkey. The studies of Aslam et al., [9], Khan et al. [19, 27] used time series models to forecast future’s scenario of COVID-19 in Pakistan. The results of Aslam et al. [9] show increasing trend and suggested eightfold increase in recoveries and an increase of 2.7 times in new cases in the end of May 2020. However, the 10 days ahead forecast of Khan et al. [19] showed that the maximum number of new cases, maximum deaths and maximum recovered cases will be 9,334, 210 and 4,761, respectively, in Pakistan. The results of this study were more realistic as compared to that of Aslam et al. [9] in terms of number of cases. The findings of Carcione et al. [6] suggested that the ending phase of COVID-19 would be in the middle of August 2020 in the Italian region of Lombardy. Fanneh et al. [28] forecasted infections, fatalities and recoveries about COVID-19 in sub-Saharan region of Africa and concluded that the infections number will be 141,733 and 986,059 in the next 30 and 60 days, respectively. The results of the current study can provide valuable information on infections, fatalities and recoveries about COVID-19 in Algeria. Therefore, it is suggested to concerned departments and agencies to consider the findings of this study as guidelines while developing future’s plans regarding COVID-19. It is to mention that this study is adapted only for short-term forecasting and based only on official reported numbers knowing that the real numbers of cases are much higher. At last one of the limitation of our model is that it does not include preventive measures and consider them as stable in the forecasted period.

## Conclusions

Time series VAR model has been used to describe the data generating process of COVID-19 (daily new infections, fatalities and recoveries) in Algeria. VAR model was used for 20 days ahead forecast and it was noted that the forecast results of all these variables follow the trend of observed data reasonably. For daily new infections, it was noted that the minimum number of new cases will be 63 [CI = − 89 to 214] in the upcoming 20 days. The maximum number of new cases will be 147 [CI = 108–186]. The minimum number of forecasted fatalities will be 8 [CI = − 1 to 17] while maximum number will be 12 [CI = 4–20]. The forecast results suggest also that there will be 40 [CI = − 106 to 185] and 142 [CI = 4–239] minimum and maximum number of recovered cases, respectively. The results suggest that the average number of recoveries is less than the average number of infections, and therefore, probably the number of infected people will increase in future. The findings of this study may be helpful for health, education and other concerned departments in Algeria.

## Data Availability

Not applicable.

## Abbreviations

ARIMA: Autoregressive integrated moving average
COVID-19: Coronavirus disease 2019
LSTM: Long short-term memory networks
SIR: Susceptible–infectious–recovered
SEIR: Susceptible–exposed–infectious–recovered
TSMs: Time series models
VAR: Vector autoregressive
